# Down-regulation of multiple CDK inhibitor *ICK/KRP* genes promotes cell proliferation, callus induction and plant regeneration in *Arabidopsis*

**DOI:** 10.3389/fpls.2015.00825

**Published:** 2015-10-13

**Authors:** Yan Cheng, Han Liu, Ling Cao, Sheng Wang, Yongpeng Li, Yuanyuan Zhang, Wei Jiang, Yongming Zhou, Hong Wang

**Affiliations:** ^1^National Key Laboratory of Crop Genetic Improvement, College of Plant Science and Technology, Huazhong Agricultural UniversityWuhan, China; ^2^Department of Biochemistry, University of Saskatchewan, SaskatoonSK, Canada

**Keywords:** *Arabidopsis thanliana*, cell cycle, cyclin-dependent kinase, CDK inhibitor, *ICK/KRP*, cell proliferation, callus induction, plant regeneration

## Abstract

The ICK/KRP cyclin-dependent kinase (CDK) inhibitors are important plant cell cycle regulators sharing only limited similarity with the metazoan CIP/KIP family of CDK inhibitors. Information is still limited regarding the specific functions of different *ICK/KRP* genes *in planta*. We have shown previously that down-regulation of multiple CDK inhibitor *ICK/KRP* genes up-regulates the E2F pathway and increases cell proliferation, and organ and seed sizes in *Arabidopsis*. In this study, we observed that the quintuple *ick1/2/5/6/7* mutant had more cells in the cortical layer of the root apical meristem (RAM) than the wild type (Wt) while its RAM length was similar to that of the Wt, suggesting a faster cell cycle rate in the quintuple mutant. We further investigated the effects of down-regulating *ICK* genes on tissue culture responses. The cotyledon explants of *ick1/2/5/6/7* could form callus efficiently in the absence of cytokinin and also required a lower concentration of 2,4-D for callus induction compared to the Wt plants, suggesting increased competence for callus induction in the mutant. In addition, the quintuple *ick* mutant showed enhanced abilities to regenerate shoots and roots, suggesting that increased competence to enter the cell cycle in the quintuple mutant might make it possible for more cells to become proliferative and be utilized to form shoots or roots. These findings indicate that CDK activity is a major factor underlying callus induction and increased cell proliferation can enhance *in vitro* organogenesis.

## Introduction

Cell division is fundamental to plant growth, development, and reproduction. In eukaryotes, cyclin-dependent kinases (CDKs) control cell division cycle, and their activities are in turn modulated by different factors (Morgan, 1997). Among them, CDK inhibitors are crucial negative regulators which inhibit CDK activity through direct protein binding. CDK inhibitor genes were initially identified in mammalian and yeast (Sherr and Roberts, 1995). The first plant CDK inhibitor gene *ICK1* (Inhibitor of CDK) was discovered in *Arabidopsis* (Wang et al., 1997) and there are seven *ICK* genes (also refered to as *KRPs*) in *Arabidopsis* (De Veylder et al., 2001). To date, *ICK/KRP* genes have been identified from different plant species such as tobacco (Jasinski et al., 2003), maize (Coelho et al., 2005), rice (Barrôco et al., 2006), tomato (Bisbis et al., 2006), apple (Malladi and Johnson, 2011), and avocado (Sabag et al., 2013).

Tissue culture and plant regeneration from explants have many different applications. When proper stimuli are given, somatic plant cells may form adventitious embryos, root, or shoots (De Klerk et al., 1997). Plant regeneration usually takes one of the two pathways: somatic embryogenesis and organogenesis (Davey and Anthony, 2010). The plant regeneration process can be divided conceptually into the following three phases: (1) dedifferentiation, during which the cells acquire competence to respond to the induction stimuli; (2) induction, during which the competent cells are induced to enter particular morphologic pathways; and (3) realization, during which the calli undergo morphological differentiation and development (see review by Duclercq et al., 2011). The organogenesis pathway is more often the route encountered in micropropagation, haploid production and plant transformation (Duclercq et al., 2011). During *in vitro* organogenesis, callus induction is followed by shoot and root regeneration. It has been demonstrated in a wide range of plant species that generally a high cytokinin (CK) to auxin ratio induces shoot organogenesis, whereas a low ratio results in root development (Sangwan and Sangwan-Norreel, 1990; De Klerk et al., 1997; Davey and Anthony, 2010). In addition to the exogenous plant hormones, other conditions such as nutrient concentrations, sugar sources, and induction time on culture media can also affect the frequency of plant regeneration (Christianson and Warnick, 1983).

In recent years, considerable progress has been made in understanding the developmental events during *de novo* organogenesis and its underlying molecular mechanisms. Certain genes involved in shoot and root organogenesis processes have been identified (see reviews: Duclercq et al., 2011; Motte et al., 2014). Most of these genes are involved in auxin and CK pathways or shoot meristem maintenance. In addition, *ESR1* and *ESR2* encoding transcriptional factors of the *AP2/ERF* family, are identified as enhancers of shoot regeneration in *Arabidopsis* (Banno et al., 2001; Ikeda et al., 2006), and *CUP-SHAPED COTYLEDON1 (CUC1)*, *CLAVATA3/EMBRYO SURROUNDING REGION-RELATED PEPTIDE (CLE2)* and *GCN5-related N-acetyltransferase 1 (GNAT1)* were identified as *ESR1* up-regulated genes (Matsuo et al., 2009), while *CUC1*, *Cyclin D1;1* and *ARABIDOPSIS PHOSPHOTRANSMITTER 6 (AHP6)* were identified as *ESR2* up-regulated genes (Ikeda et al., 2006), which might also be involved in shoot regeneration.

Cell division is a prerequisite to both callus induction and shoot/root regeneration during organogenesis. Although there is considerable amount of knowledge on the functions of cell cycle regulators in the cell cycle, relative little is known about their involvement *in vitro* plant regeneration. Several previous studies showed that cell cycle regulators can affect callus induction. Overexpression of a D-type cyclin has been shown to increase callus induction frequency and callus growth rate in *Arabidopsis* (Riou-Khamlichi et al., 1999; Cockcroft et al., 2000). In rice, inducible expression of a rice CDK-activating kinase also increases callus induction of tobacco leaf explants (Yamaguchi et al., 2003).

In a previous study, we have reported the effects of down-regulating ICK/KRP CDK inhibitors on plant growth and development using a series of *ick* mutants (Cheng et al., 2013a). The multiple *ick* mutants particularly the quintuple mutant had increased CDK activity, up-regulated E2F-RB pathway and enhanced cell proliferation. In this study, we investigated the effects of *ICK* down-regulation on root cell proliferation and further on callus induction and plant regeneration.

## Materials and Methods

### Plant Materials and Growth Conditions

The T-DNA insertion lines for the five *Arabidopsis ICK*/*KRP* genes used in this study have been described previously (Cheng et al., 2013a). For plants in soil, *Arabidopsis* plants were grown at 21°C under 16 h/8 h day/night photoperiod in a plant growth room. For seedlings in plates, seeds were sterilized as described (Valvekens et al., 1988) and sowed on ½-strength solid MS medium (1/2 MS, 1% sucrose, 0.7% agar, pH 5.8). The plates were placed vertically in the tissue culture room with the temperature of 22°C and photoperiod of 16 h/8 h day/night.

### Root Length and Root Meristem Size Analysis

The seeds of wild type (Wt) and *ick1/2/5/6/7* quintuple mutant were sterilized and sowed on square plates containing ½-strength solid MS medium. The plates were placed vertically in the plant tissue culture room. Everyday from 2 to 6 days after germination (DAG), 16–25 seedlings from each line were removed from the plates for root length and meristem size analyses. The root length was measured with a ruler. To measure root meristem size, the root was removed and immersed in Hoyer’s solution chloral hydrate/water/glycerol (3:0.8:0.4) on a glass slide. After 30 min treatment, the slide was covered with a coverslip. The root meristem was observed with DIC (differential interference contrast) under a microscope (Nikon ECLIPSE 80i). The root meristem size was represented by the number of meristematic cortex cells, which was counted as described (Casamitjana-Martíneznez et al., 2003; Ioio et al., 2007). The length of cortex cells in the mature zone was determined at 6 DAG. For each root, an image was taken and cell length measured for 6–10 cortex cells along the mature zone using Image J (http://rsb.info.nih.gov/ij).

### Callus Induction and Growth Analyses

Sterilized Wt and mutant *Arabidopsis* seeds were sowed on square plates containing solid 1/2 MS medium. Seven days after sowing, cotyledons were cut into explants of approximately 4 mm × 4 mm in size. The explants were placed onto 1/2 MS medium containing 0.2 mg/ml 2,4-D (2,4-dichlorophenoxy-acetic acid) or containing both 0.2 mg/ml 2,4-D and 0.2 mg/ml 6-BA (6-benzylaminopurine) solidified with 0.7% agarose. Ten plates for each treatment (about 15 explants in each plate) were used for callus induction and growth analyses. Every the seventh day, the explants were examined to obtain the frequency of explants with callus. Then, the explants were transferred to a fresh plate containing the same medium. The weight of the plate before and after the transfer was measured and the average weight of calli was obtained [=(weight of plate after transfer – weight of plate before transfer)/number of the calli].

To determine the minimal 2,4-D concentration for callus induction, root segments, and excised cotyledons were incubated on ½-strength solid MS medium supplemented with different concentrations of 2,4-D. (for the first batch: 0 mg/L, 0.1mg/L, 0.15 mg/L, and 0.2 mg/L; for the second batch: 0.005 mg/L, 0.01 mg/L, 0.02mg/L, 0.03 mg/L, 0.04 mg/L, 0.05 mg/L). Callus induction frequency was obtained after 10 and 20 days of culture for root explants and after 20 days for cotyledon explants. Fresh weigh was obtained after 20 days of culture. For each treatment, 5–6 plates with 32 root or cotyledon explants in each plate were used.

### Root and Shoot Regeneration Analysis

Sterilized Wt and mutant seeds were sowed on square plates containing ½-strength MS solid medium, with the plates placed vertically in the tissue culture room. Seven days after sowing, excised roots were cut into 3–5 mm segments and transferred onto callus induction medium (CIM) containing Gamborg’s B5 salt and vitamins (Gamborg et al., 1968), 2% sucrose, 0.5 g/L MES, 0.48 mg/L 2,4-D, 0.043 mg/L kinetin (KT), and 0.7% agarose. After 7 days of culture on CIM, the root explants with callus were transferred either onto shoot induction medium (SIM) containing Gamborg’s B5 and vitamins, 2% sucrose, 0.5 g/L MES, 1 mg/L isopentenyladenine (2-ip), 0.15 mg/L indole-3-acetic acid (IAA), and 0.7% agarose for regenerating shoots, or onto root induction medium (RIM) containing Gamborg’s B5 and vitamins, 2% sucrose, 0.5 g/L MES, 0.87 mg/L IAA and 0.7% agarose for regenerating roots (Yasutani et al., 1994). For shoot regeneration analysis, each line had 9–10 SIM plates with each plate having ∼50 explants. After 30 days of culture, the calli were surveyed to obtain the frequency of shoot regeneration for each plate.

To determine shoot and root regeneration in different *ick* mutant lines, calli were first induced from root explants as described above. The explants with callus were then transferred onto SIM for shoot induction or RIM for root induction, with 9–10 plates for each treatment. For shoot induction each plate had ∼40 cultured root explants (root explants with callus), while for root induction each plate about 50 cultured root explants transferred from the CIM. After 20 days of culture on SIM or RIM, the number of calli with regenerating shoots was counted and shoot regeneration frequency obtained for each plate. For root regeneration, we determined the frequency of explants with root induction (=number of calli with regenerating roots/total number of calli). Since the number of roots on each callus varied greatly, we grouped the explants into four categories: (1) no root, (2) 1–5 roots, (3) 6–10 roots and (4) more than 10 roots. The percentages of the four categories were calculated for each line.

### RNA Extractions and Real-time PCR

*Arabidopsis* total RNA was isolated using TRIzol reagent (Invitrogen) according to manufacturer’s instructions. First-strand cDNA synthesis and real-time PCR analysis were performed as described previously (Zhang et al., 2015). The primers for real-time PCR are listed in Supplementary Table S1.

### Statistical Analysis

The Mann–Whitney two-tailed *U* test was used for analyzing the differences in callus induction rates from cotyledon explants and root induction rates from root explants. Fisher’s least significant difference (LSD) method was used for multiple comparisons of shoot regeneration rates of the Wt and mutants. For the other differences between the mutant and Wt, The Student’s *t*-test (*T*-test) method was used and the analysis was performed.

## Results

### Cell Division was Accelerated in Root Cortex Meristematic Cells of *ick1/2/5/6/7* Mutant

In our previous study, we established a series of T-DNA insertion lines in which one to five *ICK* genes were knocked out, and observed phenotypical changes in triple, quadruple and quintuple mutants. Notably the quintuple mutant *ick1 ick2 ick5 ick6 ick7* (referred to *as ick1/2/5/6/7* for shoot) has larger leaves, petals, and seeds than the Wt (Cheng et al., 2013a), suggesting that cell proliferation is promoted in the *ick1/2/5/6/7* quintuple mutant, as a result of down-regulating *ICK* genes.

**FIGURE 1:**
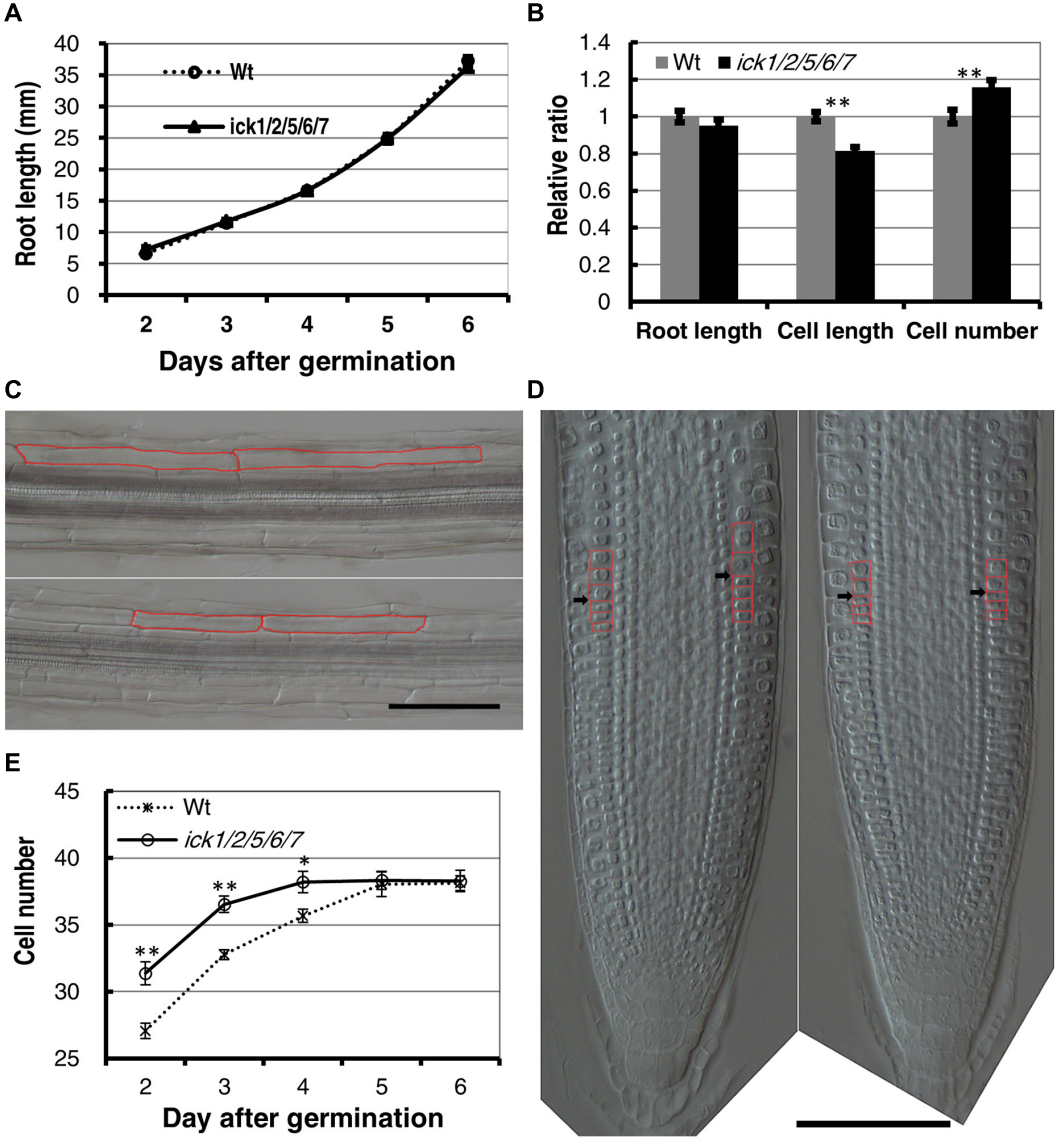
**Cell length, cell number, and root apical meristem size of wild type (Wt) and *ick1/2/5/6/7* mutant *Arabidopsis* plants. (A)** Primary root length of the Wt and *ick1/2/5/6/7* quintuple mutant at 2–6 days after germination (DAG). Each datum point represents the average of 16–25 seedlings. **(B)** Relative root length, cortex cell length and cortex cell number of the mature zone of the Wt and *ick1/2/5/6/7* mutant plants at 6 DAG (relative ratio = value of mutant/value of Wt). For root length and cell length analyses, 20–22 seedlings were used. For cell length analysis, at least six cells in the mature zone were measured for each root tip. **(C)** Differential interference contrast (DIC) images showing the cortex cells of the Wt (upper) and *ick1/2/5/6/7* quintuple mutant (lower) roots. Two cortex cells of each line are marked in red. **(D)** Longitudinal view of root tips of 6-day-old Wt and *ick1/2/5/6/7* quintuple (right) plants. The root tips were placed on a glass slide, covered with a coverslip, and viewed under a DIC microscope. The cortex cells around the transition zone are marked in red, and black arrowheads indicate the boundary between meristem and elongation zone. **(E)** Meristem size of Wt and *ick1/2/5/6/7* quintuple mutant plants at 2-6 DAG. The root meristem is expressed as the number of cortex cells in a file extending from the quiescent center (QC) to the first elongated cell. The Student’s *t*-test was used for analyzing the difference between the Wt and mutant [error bar = SE (standard error); ^∗^*P* < 0.05, ^∗∗^*P* < 0.01]. Each datum point represents the average of 16–25 seedlings. Bars = 100 um.

To determine more specifically how cell proliferation is affected in the mutant, we examined cell production in the root. First, we analyzed the root growth of the *ick1/2/5/6/7* mutant and Wt plants from 2 to 6 days after seed plating. As shown in **Figure 1A**, the primary root length for *ick1/2/5/6/7* quintuple mutant was very similar to that of the Wt. We then investigated the cortex cells in the mature zone of the roots at 6-day stage after germination (DAG). The length of cortex cells in different positions along each root was measured, and the total cortex cell number estimated based on the root length and average cortex cell length. The results showed that the average length of the cortex cells in the mature zone of *ick1/2/5/6/7* quintuple mutant was reduced compared with that of Wt (137.7 ± 3.6 um compared to 168.9 ± 4.1 um in Wt). Since the mutant and the Wt lines had a similar root length (36.3 ± 3.9 mm and 36.3 ± 3.2 mm at 6 DAG, respectively), the total cell number in a cortex cell file along the mature zone of *ick1/2/5/6/7* quintuple mutant (264.0 ± 8.8) was significantly higher than that of the Wt (229.1 ± 8.2; **Figures 1B,C**; **Figure 1B** shows the relative ratio of the mutant to Wt).

To determine whether the quintuple mutant has a larger root meristem, we performed a time-course analysis on root meristem size following Ioio’s method (Ioio et al., 2007). In this assay, the root-meristem size was expressed as the number of cortex cells in a file extending from the quiescent center (QC) to the first elongated cell (**Figure 1D**). We found that the roots of *ick1/2/5/6//7* quintuple mutant and Wt had a similar final root meristem size. However, the *ick1/2/5/6/7* quintuple mutant reached the final size 4 DAG, while the Wt reached this final size 5 DAG (**Figure 1E**), suggesting an accelerated rate of cell division and reduced cell elongation in the mutant. These results imply that more cells in the cortex of the quintuple mutant are likely due to a faster cell production rate, instead of a larger root meristem.

### Down-regulation of Five *ICKs* Increased Callus Induction

**FIGURE 2:**
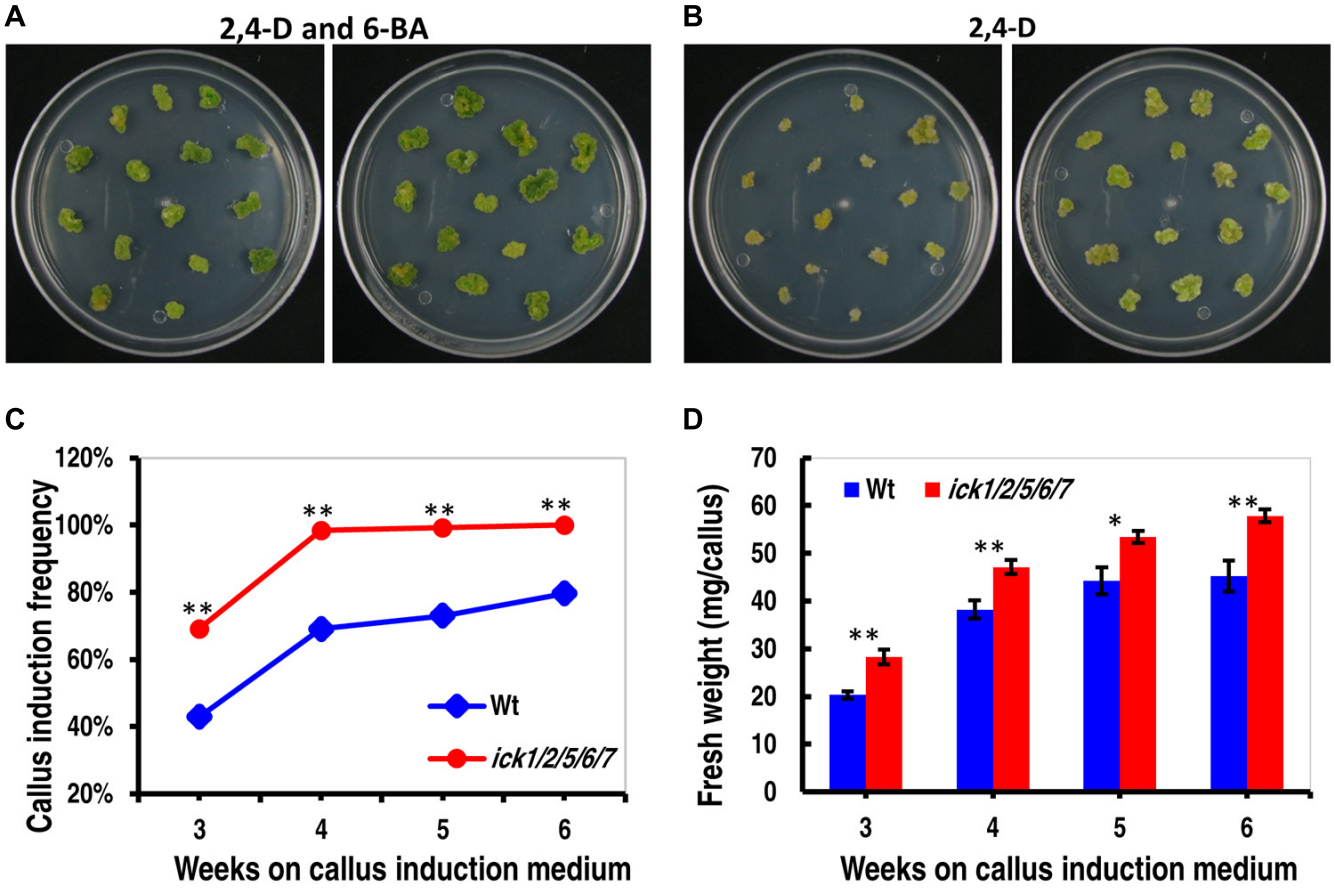
**Callus induction from cotyledon explants and callus growth of Wt and *ick1/2/5/6/7* plants. (A,B)** Representative plates showing callus induction from cotyledon explants of Wt (left) and *ick1/2/5/6/7* mutant (right) in the presence of 2,4-D and 6-BA **(A)** or 2,4-D alone **(B)**. Similar results were obtained in three independent experiments. In each experiment, 10 plates were used for each treatment, with each plate having around 15 explants. The data from one experiment are shown here. **(C)** Callus induction frequency of the Wt and *ick1/2/5/6/7* mutant explants on culture medium containing 2,4-D alone. The difference in the induction rate between the Wt and mutant at each time point was compared by Mann–Whitney *U* test. **(D)** Fresh weight of calli from the Wt and *ick1/2/5/6/7* mutant cotyledon explants after 3–7 weeks of culture on culture medium containing both 2,4-D and 6-BA. The differences between the Wt and mutant were analyzed by Student’s *t*-test (error bar = SE; ^∗^*P* < 0.05, ^∗∗^*P* < 0.01).

To further understand the impact of *ICK* down-regulation, we examined tissue culture responses since cell proliferation is critical for callus and plant regeneration. Cotyledon explants of both Wt and *ick1/2/5/6/7* mutant produced calli efficiently on 1/2 MS medium containing both 0.2 mg/ml 2,4-D and 0.2 mg/ml 6-BA. On 1/2 MS medium containing 0.2 mg/ml 2,4-D, however, there was a higher frequency of callus induction for the mutant explants (**Figures 2A,B**). For instance, 98.4% of the *ick1/2/5/6/7* mutant explants produced calli, compared 69.1% for the Wt (**Figure 2C**). In addition, the calli of quintuple mutants were much larger with lightly greenish color, while those of the Wt yellower and smaller (**Figure 2B**). These results indicate that down-regulation of the five *ICK* genes enhances callus formation and reduces CK requirement for callus induction.

To determine callus growth rate, the explants were transferred to fresh callus induction plates every week, and the callus growth was obtained by weighing the plate immediately after the transfer and on the seventh day of culture. As shown in **Figure 2D** and Supplementary Figure S1, the calli of *ick1/2/5/6/7* grew faster than those of Wt in the presence of 6-BA and 2,4-D or 2,4-D only. Those results indicate that down-regulation of the five *ICK* genes also enhances callus growth.

### Auxin Dependency for Callus Induction was Decreased in the *ick1/2/5/6/7* Mutant

**FIGURE 3:**
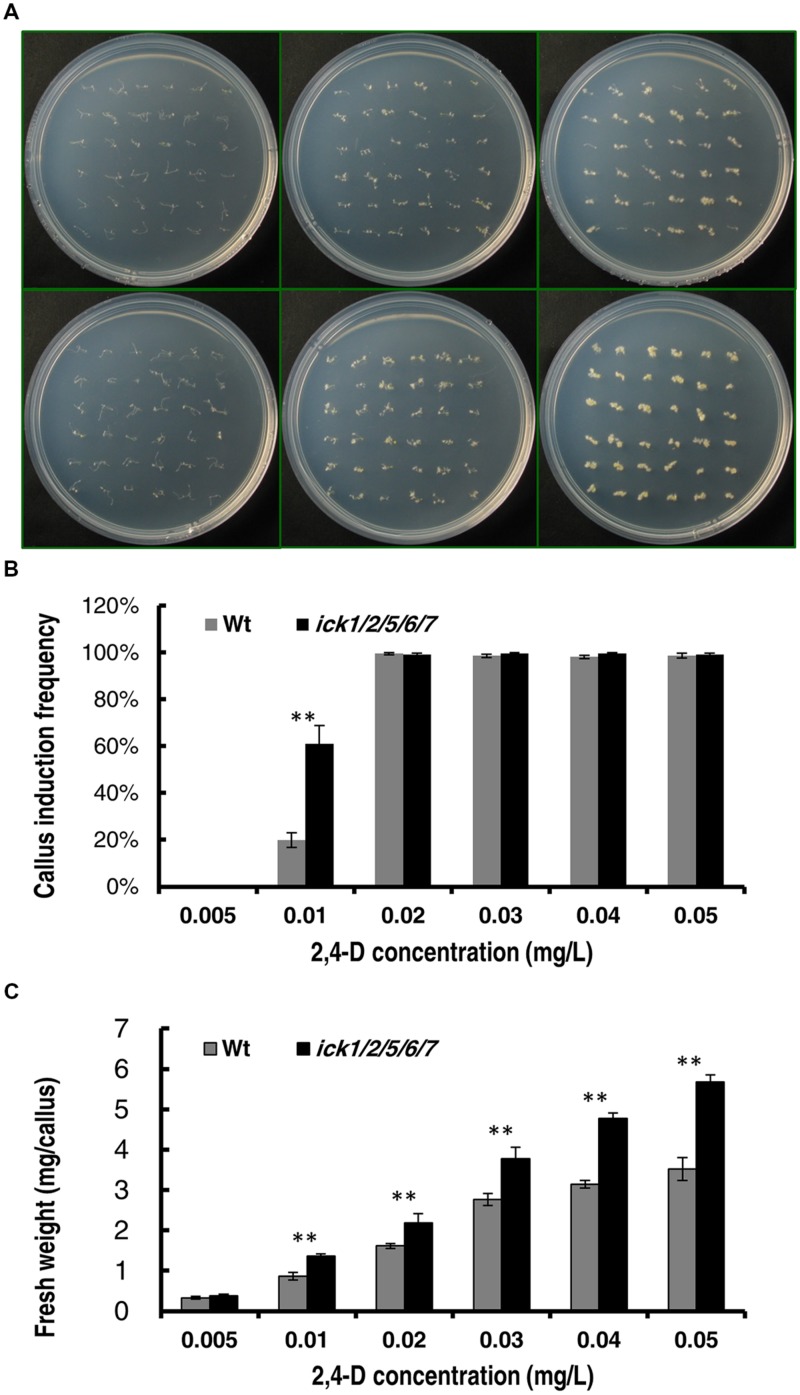
**Callus induction from root explants of the Wt and *ick1/2/5/6/7* mutant at different 2,4-D concentrations**. Root segments were cultured on 1/2 MS medium containing the indicated concentrations of 2,4-D for 20 days. **(A)** Representative plates showing the root explants of Wt (the upper row) and *ick1/2/5/6/7* mutant (the lower row) on 1/2 MS medium containing 0.05, 0.01, and 0.05 mg/L (the first–third column, respectively) 2,4-D. **(B)** Callus induction rate of root explants of Wt and *ick1/2/5/6/7* mutant after 20 days of culture. **(C)** Fresh weight of root explants with callus of Wt and *ick1/2/5/6/7* after 20 days of culture. The bars show the mean values of 4–5 plates. Student’s *t*-test was used for analyzing the differences in induction rate and fresh weight between the Wt and mutant (error bar = SE; ^∗∗^*P* < 0.01).

To further confirm that *ICK* down-regulation reduces auxin requirement for callus induction, we determined the minimal 2,4-D concentration for callus induction from root explants of both Wt and mutant plants. In this assay, the root segments (about 5 mm in length) were incubated on the 1/2 MS medium supplemented with different concentrations of 2,4-D. We first used 2,4-D concentrations of 0, 0.05, 0.1, 0.15, and 0.2 mg/L. Neither the Wt nor the quintuple mutant showed callus induction on 1/2 MS medium without 2,4-D after 20 days of culture; whereas, on the culture plates containing 0.05, 0.1, 0.15, or 0.2 mg/L 2,4-D, almost all segments of both lines generated calli (Supplementary Figures S2A,B). We then used a series of lower 2,4-D concentrations of 0.005, 0.01, 0.02, 0.03, 0.04, and 0.05 mg/L. The root explants of both lines produced no callus at 0 and 0.005 mg/L 2,4-D, and almost all root explants of both lines produced calli at 0.02, 0.03, 0.04, and 0.05 mg/L 2,4-D after 20 days of culture (**Figure 3A**). At 0.01 mg/ml 2,4-D, the callus induction frequency of *ick1/2/5/6/7* mutant (61.1%) was significantly higher than that of the Wt (19.9%; **Figure 3B**). This observation suggests that the *ick1/2/5/6/7* mutant needs a lower concentration of auxin for callus induction compared to Wt. Moreover, for the 2,4-D concentrations at which the callus induction frequencies of the two lines were comparable, the fresh weights of calli from the mutant root explants were significantly higher than those of Wt (**Figure 3C** and Supplementary Figure S2C), consistent with observation made with the cotyledon explants.

**FIGURE 4:**
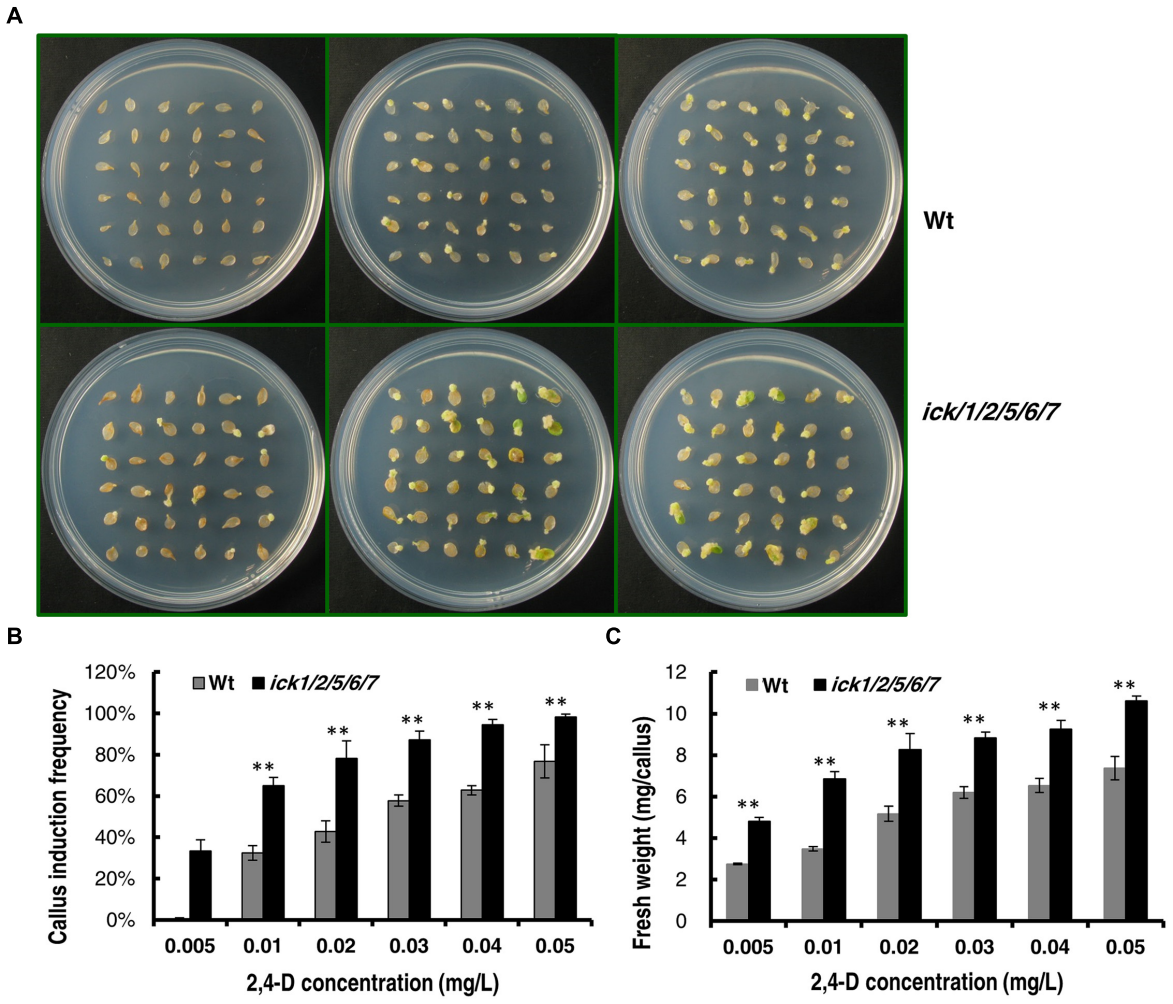
**Callus induction from cotyledon explants of the Wt and *ick1/2/5/6/7* mutant at different 2,4-D concentrations**. The cotyledons were excised form 7-day-old seedlings and cultured on 1/2 MS medium for 20 days. **(A)** Representative plates showing the cotyledon explants of Wt (the upper row) and *ick1/2/5/6/7* mutant (the lower row) on 1/2 MS medium containing 0.005 mg/L, 0.02g/L, 0.04 mg/L (the first–third column) 2,4-D. **(B)** Callus induction rate of the cotyledon explants of *ick1/2/5/6/7* and Wt on the indicated media. **(C)** Fresh weight of the cotyledon explants with callus of *ick1/2/5/6/7* and Wt on the indicated media. The bars show the mean induction rates of 4–5 plates. Student’s *t*-test was used for analyzing the differences in induction rate and fresh weight between the Wt and mutant (error bar = SE; ^∗∗^*P* < 0.01).

We also determined the minimal 2,4-D concentration required for callus induction from cotyledonary petiole of both lines. When the 2,4-D concentration was higher than 0.05 mg/ml, almost all of the cotyledonary petiole explants of *ick1/2/5/6/7* produced calli (Supplementary Figures S3A,B). When the 2,4-D concentration was lower than 0.1 mg/ml, the callus induction frequency of the *ick1/2/5/6/7* mutant was significantly higher than that of the Wt (**Figures 4A,B** and Supplementary Figures S3A,B). At 0.005 mg/ml 2,4-D, 33% of *ick1/2/5/6/7* cotyledonary petioles showed callus induction, while no cotyledonary petioles of the Wt did (**Figures 4A,B**). Also, at various concentrations of 2,4-D the calli of the *ick1/2/5/6/7* mutant were significantly larger than those of Wt (**Figure 4C** and Supplementary Figure S3C), further confirming that the callus of *ick1/2/5/6/7* mutant was growing faster than the Wt callus.

### Shoot and Root Regeneration was Enhanced in *ick1/2/5/6/7* Mutant

In addition to callus induction, shoot and root regeneration is another important aspects of plant tissue culture. Thus, we investigated the ability of the root explants to regenerate shoots. Root explants (about 5 mm segments) were first cultured on CIM. After 7 days, explants with callus were transferred to SIM. As shown in Supplementary Figure S4, the shoot regeneration frequency of *ick1/2/5/6/7* quintuple mutant was significantly higher than that of Wt after 30 days of culture. Also, each of the explants of *ick1/2/5/6/7* quintuple mutant on average regenerated more roots than that of Wt. Those results indicate that the *ick1/2/5/6/7* quintuple mutant has a stronger ability to regenerate shoots and roots.

### Disruption of *ICK* Genes Additively Promoted Shoot and Root Regeneration

**FIGURE 5:**
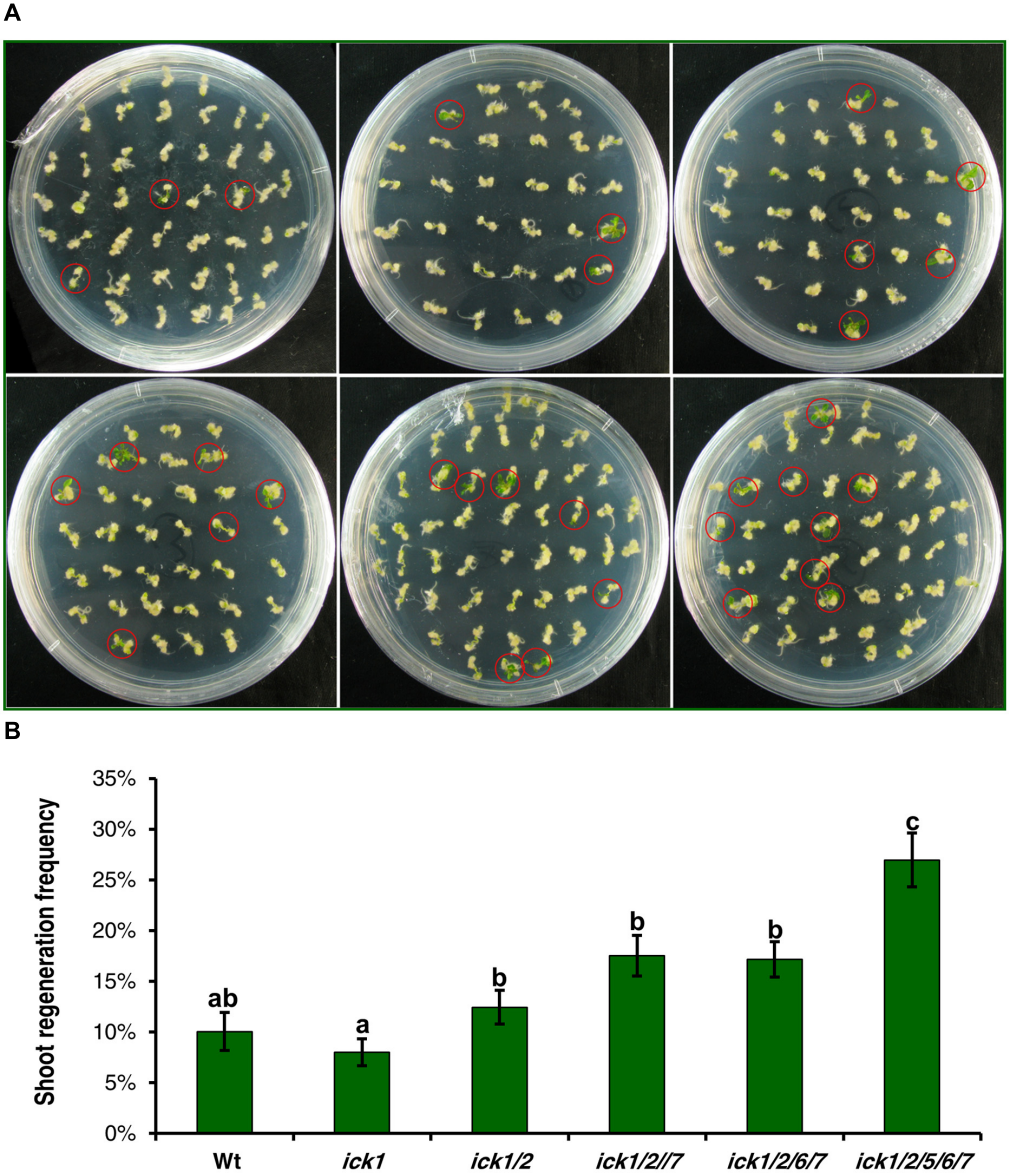
**Shoot regeneration from root-derived calli of Wt and various *ick* mutant plants**. Root explants were cultured on callus induction medium (CIM) first. After 7 days, root explants with callus were transferred onto shoot induction medium (SIM) and cultured for 30 days. For each line, 9–10 plates were used with each plate having about 40 explants. **(A)** Representative plates showing shoot regeneration on SIM. The plates in the first row are: Wt (left), *ick1* (middle), *ick1/2* (right), and in the second row, *ick1/2/7* (left), *ick1/2/6/7* (middle), and *ick1/2/5/6/7* (right). The red circles mark the calli with regenerating shoots. **(B)** Frequency of shoot regeneration in Wt and *ick* mutants (Mean ± SE). The significant differences among different lines were analyzed by Fisher’s least significant difference (LSD) method, and are indicated by different lowercase letters (*P* < 0.05).

Our previous results on a series T-DNA mutants showed that the effects from down-regulating *ICK* genes become more evident as more *ICK* genes are disrupted (Cheng et al., 2013a). Therefore, we selected a series of single, double, triple, quadruple, and quintuple mutants to determine whether such an additive effect of multiple loci also exists for shoot and root regeneration. For this analysis, root explants with callus were transferred onto SIM or RIM (RIM) for shoot or root regeneration. After culturing on SIM for 20 days, the single *ick1* mutant had a similar frequency of calli with regenerating shoots to the Wt, and there was a trend of increasing regeneration frequency from the single to the quintuple mutant (**Figure 5A**). Although the differences among Wt, *ick1/2*, *ick1/2/7*, *ick1/2/6/7* did not reach a significant level, the quintuple *ick1/2/5/6/7* mutant with the highest frequency showed significant differences from the other lines (**Figure 5B**).

Calli were also cultured on RIM for 20 days, although most calli of all the lines produced roots, the calli of *ick1/2/6/7* and *ick1/2/5/6/7* mutants had visibly more roots than Wt and *ick1*, *ick1/2* and *ick1/2/7* mutants (Supplementary Figure S5A). We first compared the root regeneration rate of the six lines. The root regeneration frequencies of *ick1/2/6/7* and *ick1/2/5/6/7* were significantly higher than those of other lines (Supplementary Figure S5B). To better characterize the number of roots per callus, the calli were grouped into four categories, with 0, 1–5, 6–10, and more than 10 roots per callus, respectively. The calli of *ick1/2/6/7* and *ick1/2/5/6/7* mutants had significant more roots than the calli of other lines (Supplementary Figure S5C). Those results indicate that down-regulation of *ICK* genes additively enhances both the abilities for shoot and root regeneration.

### E2F-dependent Genes and Shoot Regeneration Related Genes were Mostly Up-regulated During Shoot Regeneration of *ick1/2/5/6/7*

**FIGURE 6:**
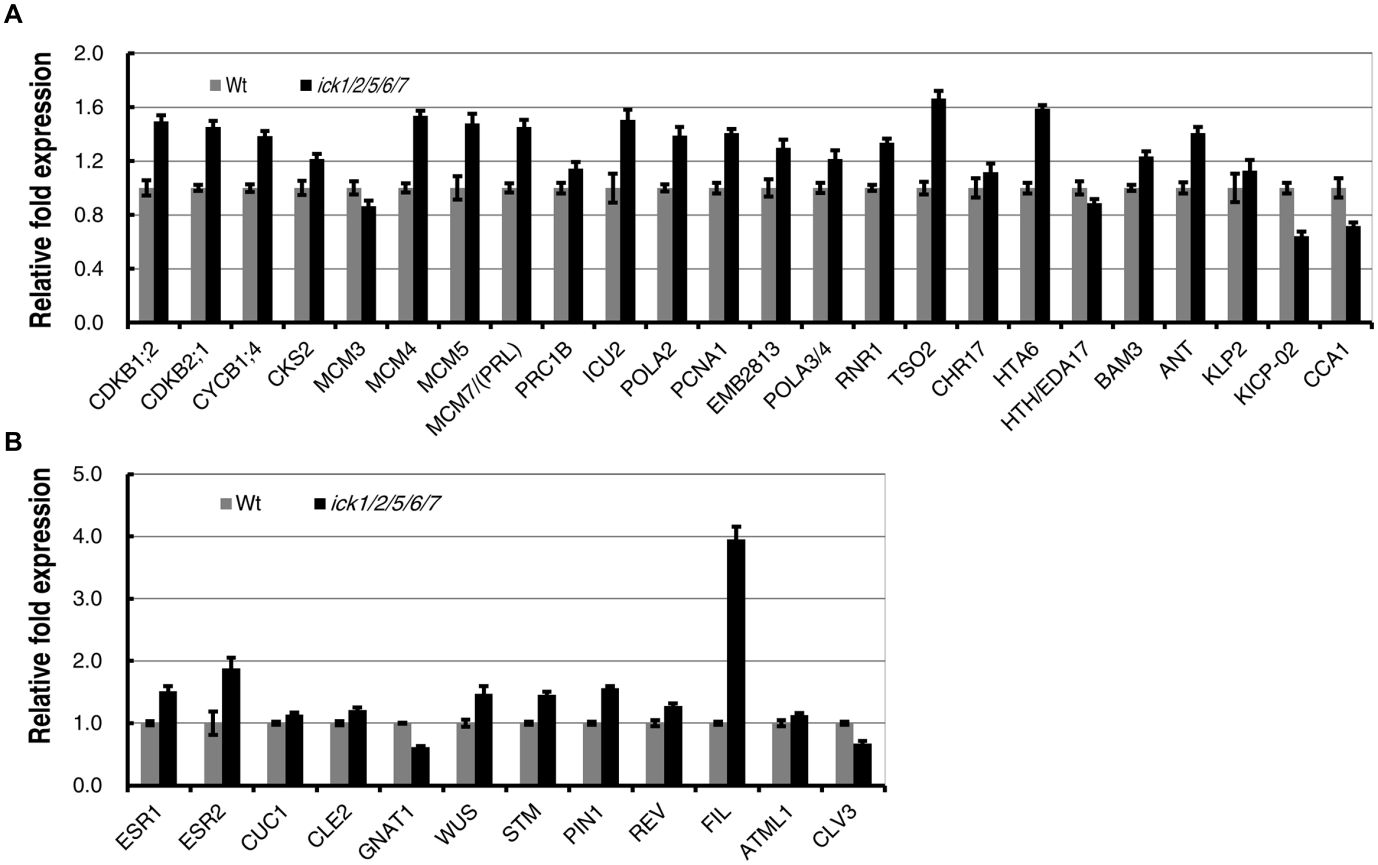
**Expression of E2F-dependent and shoot regeneration related genes during *in vitro* shoot regeneration of the Wt and *ick1/2/5/6/7* mutant**. Root explants were cultured on CIM for 7 days and then transferred onto SIM. Total RNA was extracted after 7 days of culture on SIM. Each value on the vertical axes indicates a relative fold expression level calculated by scaling to the *Ubq10c* transcript level. Data present the average of four biological repeats and error bars indicated standard errors. Each biological repeat has four technical replicates. **(A)** E2F-dependent genes. **(B)** Shoot regeneration related genes.

In our previous studies, we have demonstrated that the E2F-dependent genes are up-regulated in the *ick1/2/5/6/7* seedlings (Cheng et al., 2013a). The expression levels of the same group of E2F-dependent genes that function in cell cycle, DNA synthesis, chromatin structure, metabolism, plant development, cell structure, and light signaling/photosynthesis (Ramirez-Parra et al., 2003; de Jager et al., 2009) were analyzed in the SIM incubated callus of *ick1/2/5/6/7* mutant and Wt. Of the 24 genes analyzed, 20 had a higher level of expression in the mutant, with 17 of them having a relative fold expression higher than 1.19 (equal to the Log2 value of 0.25). The four down-regulated genes are *MCM3*, *HTH/EDA17*, *KICP-02*, and *CCA1* (**Figure 6A**). This result suggests that E2F-pathway was also enhanced in the callus of *ick1/2/5/6/7* mutant as observed in the seedlings (Cheng et al., 2013a).

It has been demonstrated that a number of genes were up-regulated during *de novo* shoot regeneration (Duclercq et al., 2011; Motte et al., 2014). To investigate whether the increased shoot regeneration ability of *ick1/2/5/6/7* mutant is accompanied by the up-regulation of these *de novo* regeneration related genes, a group of 12 genes were selected and their expression levels analyzed in the calli cultured on SIM for shoot regeneration. Of the 12 genes, *ESR1* and *ESR2* are known as the enhancers of shoot regeneration (Banno et al., 2001; Matsuo et al., 2009), *CUC1*, *CLE2*, and *GNAT1* are induced by ESR1 (Matsuo et al., 2009), and *WUS*, *STM*, *PIN1*, *REV*, *FIL*, *ATML1*, and *CLV3* are up-regulated during *de novo* shoot formation (Gordon et al., 2007; Duclercq et al., 2011). Among these 12 genes, 10 were up-regulated in the calli of *ick1/2/5/6/7* mutant, while only 2 (*GNAT1* and *CLV3*) were down-regulated (**Figure 6B**). Interestingly, the *FIL* was highly up-regulated in the callus of *ick1/2/5/6/7* with about fourfold of expression relative to that in the Wt calli.

## Discussion

### Down-regulation of Five CDK Inhibitor Genes Promotes Cell Proliferation in Roots

Root growth is determined by the balance between cell division and cell elongation (Beemster and Baskin, 1998). The defined cortical layer development in *Arabidopsis* provides a good tool to investigate the cell proliferation rate (Ivanov and Dubrovsky, 1997). Several studies using this approach have revealed that a reduced root apical meristem (RAM) size is responsible for the observed inhibition of primary root growth under different conditions (Westet al., 2004; Ubeda-Tomás et al., 2009; González-García et al., 2011). In this study, we observed that the same number of cortex meristematic cells in *ick1/2/5/6/7* quintuple mutant generated more but smaller cells in the mature zone compared to the Wt, indicating an accelerated rate of cell division in the mutant. Previously, we have shown that CDK activity (most likely CDKA) is enhanced in the *ick* mutants (Cheng et al., 2013a), which is likely to be responsible for the increased rate of cell division in roots.

### CDK Kinase Activity is a Major Factor Underlying Callus Induction

In various plant species, callus induction depends on exogenous application of both auxin and CK. The factors underlying callus induction/repression and the genes involved in these processes have been reviewed recently by Ikeuchi et al. (2013). It has been observed that the transgenic *Arabidopsis* overexpressing a putative CK receptor *CKI1* could produce callus efficiently in the absence of CK (Kakimoto, 1996), indicating the significance of CK in callus induction. Also, leaf explants of transgenic *Arabidopsis* constitutively expressing a cell cycle regulator gene *CYCD3* could also produce calli in the absence of exogenous CK (Riou-Khamlichi et al., 1999). It has been shown that CK promotes G1/S and G2/M transitions through regulating CDK activities (Sieberer et al., 2003; Del Pozo et al., 2005). Furthermore, overexpression of rice *R2*, a CDK-activating kinase, also results in CK-independent callus induction in tobacco (Yamaguchi et al., 2003). In this study, we showed that callus could be induced in the *ick1/2/5/6/7* quintuple mutant in the absence of CK. The increased CDK activity in the *ick1/2/5/6/7* mutant must have lowered the threshold requirement for CK as well as auxin for cells in the explants to enter and progress through the cell cycle. In addition, antisense expression of *Nicto;CYCA3;2* in tobacco has been observed to impairs callus formation (Yu et al., 2003). Together, these results suggest that regulation of CDK activity is a key determinant of callus induction.

It is well known that auxin along with auxin signaling modules is required for callus formation (Fan et al., 2012; Perianez-Rodriguez et al., 2014). Part of the auxin requirement may be for up-regulating certain cell cycle genes. It has been shown that *Arabidopsis* CDKA is induced by auxin (Hemerly et al., 1993). Our results showing that knockdown of *ICK* genes also lowers the threshold requirement for auxin in terms of callus induction suggests that the effect of auxin on callus induction is at least partially through CDK.

It has been reported that transgenic *Arabidopsis* plants overexpressing two TFs genes, *HB52* and *CRF3*, exhibit spontaneous callus formation without exogenous phytohormone in some organs (Xu et al., 2012). It is not known whether CDK activity is enhanced in those transgenic plants. However, genome-wide transcriptome profiling during callus initiation has revealed the up-regulation of many cell-cycle related genes (Xu et al., 2012). Thus, it is possible that *HB52* and *CRF3* promote callus formation in the absence of exogenous hormones through these cell cycle genes.

### Increased Cell Proliferation Enhances *In Vitro* Organogenesis

In the classical scheme for plant regeneration, the explants undergo dedifferentiation to obtain pluripotency during callus induction. The calli are then transferred to SIM or RIM to induce shoots and roots, respectively. Sometimes, shoots and roots can be induced at the same time. During callus induction, founder meristem cells arise in the pericycle of root explants (Atta et al., 2009). Depending on the subsequent culture conditions, the cell fate of organ primordia is determined to be either shoot or root identity (Christianson and Warnick, 1983; Atta et al., 2009). During *in vitro* organogenesis, auxin is the main phytohormone for root organogenesis, while CK promotes shoot organogenesis (Skoog and Miller, 1957; Duclercq et al., 2011; da Rocha Correa et al., 2012). Thus, the auxin-CK crosstalk is important for organ formation and identity determination (Gordon et al., 2009; Besnard et al., 2011; Cheng et al., 2013b; Zhao et al., 2013). Motte et al. (2014) reviewed the mutants with altered regeneration phenotypes, and noticed that most of the genes are related to meristem maintenance, and auxin and CK signaling.

Our findings that the quintuple *ick* mutant with increased CDK activity showed increased shoot/root organogenesis from both the root and cotyledonary explants indicate enhanced competence for cell proliferation can also promote organogenesis. We speculate that under the auxin and CK conditions favoring organ formation, increased competence to enter the cell cycle in the quintuple mutant makes it possible for more cells to become proliferative and capable of forming shoots or roots, while under conditions favoring unorganized growth increased competence of cell proliferation makes it easier to induce callus formation. Consistent with this suggestion, application of cell cycle inhibitors during SIM incubation was shown to significantly impair organogenesis in *Arabidopsis* (Che et al., 2007). In addition, *Arabidopsis ESR1* and *ESR2*, belonging to the *AP2/EAR* family transcription factors, both could enhance shoot regeneration (Banno et al., 2001; Ikeda et al., 2006). Overexpression of *ESR2* has been shown to up-regulate cell cycle genes (Ikeda et al., 2006), suggesting that ESRs may promote shoot regeneration by up-regulating cell cycle machinery and cell proliferation.

We further observed that E2F-dependent genes and shoot regeneration related genes generally showed higher levels of expression in the *ick1/2/5/6/7* during shoot regeneration compared to Wt plants (**Figures 6A,B**) suggesting that enhanced shoot regeneration is accompanied by the up-regulation of a consort of genes involved in the regeneration process. Interestingly, among them, *FIL* was highly up-regulated in *ick1/2/5/6/7* (**Figure 6B**). Although the regulation of *FIL* expression in the quintuple mutant during shoot regeneration is unknown, it is interesting to note that *FIL* gene encodes a *YABBY* (*YAB*) family putative transcription factor that has been implicated in specifying abaxial cell identities and thus being involved in development of leaves and floral organs, and in meristem activity (Sawa et al., 1999; Lugassi et al., 2010).

## Conclusion

In this study, we have demonstrated that down-regulation of CDK inhibitor genes results in enhanced shoot/root regeneration. To date, while efficient plant regeneration system has been established in a range of plant species, other plant species remain recalcitrant. Since regulation of the cell cycle by CDK is conserved through plants and all eukaryotes, it is tempting to speculate that callus induction can be enhanced through modulating CDK activity in other plants as well. *In vitro* plant regeneration has been optimized most empirically by testing a variety of hormonal and culture conditions. The realization that CDK regulation plays a key role provides molecular means to enhance plant regeneration and possibly plant transformation for applications in different plants, particularly crop species.

## Conflict of Interest Statement

The authors declare that the research was conducted in the absence of any commercial or financial relationships that could be construed as a potential conflict of interest.

## References

[B1] Atta R. Laurens L. Boucheron-Dubuisson E. Guivarc’h A. Carnero E. Giraudat-Pautot V. (2009). Pluripotency of *Arabidopsis xylem* pericycle underlies shoot regeneration from root and hypocotyl explants grown in vitro. *Plant J.* 57 626–644. 10.1111/j.1365-313X.2008.03715.x 18980654

[B2] Banno H. Ikeda Y. Niu Q. W. Chua N. H. (2001). Overexpression of *Arabidopsis* ESR1 induces initiation of shoot regeneration. *Plant Cell* 13 2609–2618. 10.1105/tpc.13.12.2609 11752375 PMC139476

[B3] Barrôco R. M. Peres A. Droual A. M. De Veylder L. De Wolf J. Mironov V. (2006). The cyclin-dependent kinase inhibitor Orysa. KRP1 plays an important role in seed development of rice. *Plant Physiol.* 142 1053–1064. 17012406 10.1104/pp.106.087056 PMC1630760

[B4] Beemster G. T. Baskin T. I. (1998). Analysis of cell division and elongation underlying the developmental acceleration of root growth in *Arabidopsis thaliana*. *Plant Physiol.* 116 1515–1526. 10.1104/pp.116.4.1515 9536070 PMC35060

[B5] Besnard F. Vernoux T. Hamant O. (2011). Organogenesis from stem cells in planta: multiple feedback loops integrating molecular and mechanical signals. *Cell. Mol. Life Sci.* 68 2885–2906. 10.1007/s00018-011-0732-4 21655916 PMC11115100

[B6] Bisbis B. Delmas F. Joubès J. Sicard A. Hernould M. Inzé D. (2006). Cyclin-dependent kinase (CDK) inhibitors regulate the CDK-cyclin complex activities in endoreduplicating cells of developing tomato fruit. *J. Biol. Chem.* 281 7374–7383. 10.1074/jbc.M506587200 16407228

[B7] Casamitjana-Martínez E. Hofhuis H. F. Xu J. Liu C. M. Heidstra R. Scheres B. (2003). Root-specific CLE19 overexpression and the sol1/2 suppressors implicate a CLV-like pathway in the control of *Arabidopsis* root meristem maintenance. *Curr. Biol.* 13 1435–1441. 10.1016/S0960-9822(03)00533-5 12932329

[B8] Che P. Lall S. Howell S. H. (2007). Developmental steps in acquiring competence for shoot development in *Arabidopsis* tissue culture. *Planta* 226 1183–1194. 10.1007/s00425-007-0565-4 17581762

[B9] Cheng Y. Cao L. Wang S. Li Y. Shi X. Liu H. (2013a). Downregulation of multiple CDK inhibitor ICK/KRP genes upregulates the E2F pathway and increases cell proliferation, and organ and seed sizes in *Arabidopsis*. *Plant J.* 75 642–655. 10.1111/tpj.12228 23647236

[B10] Cheng Z. J. Wang L. Sun W. Zhang Y. Zhou C. Su Y. H. (2013b). Pattern of auxin and cytokinin responses for shoot meristem induction results from the regulation of cytokinin biosynthesis by AUXIN RESPONSE FACTOR3. *Plant Physiol.* 161 240–251. 10.1104/pp.112.203166 23124326 PMC3532255

[B11] Christianson M. Warnick D. (1983). Competence and determination in the process of in vitro shoot organogenesis. *Dev. Biol.* 95 288–293. 10.1016/0012-1606(83)90029-5 6825936

[B12] Cockcroft C. E. den Boer B. G. Healy J. S. Murray J. A. (2000). Cyclin D control of growth rate in plants. *Nature* 405 575–579. 10.1038/35014621 10850717

[B13] Coelho C. M. Dante R. A. Sabelli P. A. Sun Y. Dilkes B. P. Gordon-Kamm W. J. (2005). Cyclin-dependent kinase inhibitors in maize endosperm and their potential role in endoreduplication. *Plant Physiol.* 138 2323–2336. 10.1104/pp.105.063917 16055680 PMC1183418

[B14] da Rocha Correa L. Troleis J. Mastroberti A. Mariath J. Fett-Neto A. (2012). Distinct modes of adventitious rooting in *Arabidopsis thaliana*. *Plant Biol.* 14 100–109. 10.1111/j.1438-8677.2011.00468.x 21974782

[B15] Davey M. R. Anthony P. (2010). *Plant Cell Culture: Essential Methods*. Hoboken, NJ: John Wiley & Sons.

[B16] de Jager S. M. Scofield S. Huntley R. P. Robinson A. S. den Boer B. G. Murray J. A. (2009). Dissecting regulatory pathways of G1/S control in *Arabidopsis*: common and distinct targets of CYCD3; 1. E2Fa and E2Fc. *Plant Mol. Biol.* 71 345–365. 10.1007/s11103-009-9527-5 19662336

[B17] De Klerk G. J. Arnholdt-Schmitt B. Lieberei R. Neumann K. H. (1997). Regeneration of roots, shoots and embryos: physiological, biochemical and molecular aspects. *Biol. Plant.* 39 53–66. 10.1023/A:1000369309303

[B18] Del Pozo J. C. Lopez-Matas M. Ramirez-Parra E. Gutierrez C. (2005). Hormonal control of the plant cell cycle. *Physiol. Plant.* 123 173–183. 10.1111/j.1399-3054.2004.00420.x

[B19] De Veylder L. Beeckman T. Beemster G. T. Krols L. Terras F. Landrieu I. (2001). Functional analysis of cyclin-dependent kinase inhibitors of *Arabidopsis*. *Plant Cell* 13 1653–1668. 10.2307/3871392 11449057 PMC139548

[B20] Duclercq J. Sangwan-Norreel B. Catterou M. Sangwan R. S. (2011). De novo shoot organogenesis: from art to science. *Trends Plant Sci.* 16 597–606. 10.1016/j.tplants.2011.08.004 21907610

[B21] Fan M. Xu C. Xu K. Hu Y. (2012). Lateral organ boundaries domain transcription factors direct callus formation in *Arabidopsis* regeneration. *Cell Res.* 22 1169–1180. 10.1038/cr.2012.63 22508267 PMC3391013

[B22] Gamborg O. L. C. Miller R. A. Ojima K. (1968). Nutrient requirements of suspension cultures of soybean root cells. *Exp. Cell Res.* 50 151–158. 10.1016/0014-4827(68)90403-5 5650857

[B23] González-García M. P. Vilarrasa-Blasi J. Zhiponova M. Divol F. Mora-García S. Russinova E. (2011). Brassinosteroids control meristem size by promoting cell cycle progression in *Arabidopsis* roots. *Development* 138 849–859. 10.1242/dev.057331 21270057

[B24] Gordon S. P. Chickarmane V. S. Ohno C. Meyerowitz E. M. (2009). Multiple feedback loops through cytokinin signaling control stem cell number within the *Arabidopsis* shoot meristem. *Proc. Natl. Acad. Sci. U.S.A*. 106 16529–16534. 10.1073/pnas.0908122106 19717465 PMC2752578

[B25] Gordon S. P. Heisler M. G. Reddy G. V. Ohno C. Das P. Meyerowitz E. M. (2007). Pattern formation during de novo assembly of the *Arabidopsis* shoot meristem. *Development* 134 3539–3548. 10.1242/dev.010298 17827180

[B26] Hemerly A. S. Ferreira P. de Almeida Engler J. Van Montagu M. Engler G. Inzé D. (1993). cdc2a expression in *Arabidopsis* is linked with competence for cell division. *Plant Cell* 5 1711–1723. 10.1105/tpc.5.12.1711 8305869 PMC160398

[B27] Ikeda Y. Banno H. Niu Q. W. Howell S. H. Chua N. H. (2006). The ENHANCER OF SHOOT REGENERATION 2 gene in *Arabidopsis* regulates CUP-SHAPED COTYLEDON 1 at the transcriptional level and controls cotyledon development. *Plant Cell Physiol.* 47 1443–1456. 10.1093/pcp/pcl023 17056621

[B28] Ikeuchi M. Sugimoto K. Iwase A. (2013). Plant callus: mechanisms of induction and repression. *Plant Cell* 25 3159–3173. 10.1105/tpc.113.116053 24076977 PMC3809525

[B29] Ioio R. D. Linhares F. S. Scacchi E. Casamitjana-Martinez E. Heidstra R. Costantino P. (2007). Cytokinins determine *Arabidopsis* root-meristem size by controlling cell differentiation. *Curr. Biol.* 17 678–682. 10.1016/j.cub.2007.02.047 17363254

[B30] Ivanov V. B. Dubrovsky J. G. (1997). Estimation of the cell-cycle duration in the root apical meristem: a model of linkage between cell-cycle duration, rate of cell production, and rate of root growth. *Int. J. Plant Sci.* 158 757–763. 10.1086/297487

[B31] Jasinski S. Leite C. S. Domenichini S. Stevens R. Raynaud C. Perennes C. (2003). NtKIS2, a novel tobacco cyclin-dependent kinase inhibitor is differentially expressed during the cell cycle and plant development. *Plant Physiol. Biochem.* 41 667–676. 10.1016/S0981-9428(03)00082-2

[B32] Kakimoto T. (1996). CKI1, a histidine kinase homolog implicated in cytokinin signal transduction. *Science* 274 982–985. 10.1126/science.274.5289.982 8875940

[B33] Lugassi N. Nakayama N. Bochnik R. Zik M. (2010). A novel allele of FILAMENTOUS FLOWER reveals new insights on the link between inflorescence and floral meristem organization and flower morphogenesis. *BMC Plant Biol.* 10:131 10.1186/1471-2229-10-131 PMC3017777 20584289

[B34] Malladi A. Johnson L. K. (2011). Expression profiling of cell cycle genes reveals key facilitators of cell production during carpel development, fruit set, and fruit growth in apple (Malus × domestica Borkh). *J. Exp. Bot.* 62 205–219. 10.1093/jxb/erq258 20732881 PMC2993910

[B35] Matsuo N. Mase H. Makino M. Takahashi H. Banno H. (2009). Identification of ENHANCER OF SHOOT REGENERATION 1-upregulated genes during in vitro shoot regeneration. *Plant Biotechnol.* 26 385–393. 10.5511/plantbiotechnology.26.385

[B36] Morgan D. O. (1997). Cyclin-dependent kinases: engines, clocks, and microprocessors. *Annu. Rev. Cell Dev. Biol.* 13 261–291. 10.1146/annurev.cellbio.13.1.261 9442875

[B37] Motte H. Vereecke D. Geelen D. Werbrouck S. (2014). The molecular path to in vitro shoot regeneration. *Biotechnol. Adv.* 32 107–121. 10.1016/j.biotechadv.2013.12.002 24355763

[B38] Perianez-Rodriguez J. Manzano C. Moreno-Risueno M. A. (2014). Post-embryonic organogenesis and plant regeneration from tissues: two sides of the same coin? *Front. Plant Sci.* 5:219 10.3389/fpls.2014.00219 PMC4033269 24904615

[B39] Ramirez-Parra E. Fründt C. Gutierrez C. (2003). A genome-wide identification of E2F-regulated genes in *Arabidopsis*. *Plant J.* 33 801–811. 10.1046/j.1365-313X.2003.01662.x 12609051

[B40] Riou-Khamlichi C. Huntley R. Jacqmard A. Murray J. A. (1999). Cytokinin activation of *Arabidopsis* cell division through a D-type cyclin. *Science* 283 1541–1544. 10.1126/science.283.5407.1541 10066178

[B41] Sabag M. Ari G. B. Zviran T. Biton I. Goren M. Dahan Y. (2013). PaKRP, a cyclin-dependent kinase inhibitor from avocado, may facilitate exit from the cell cycle during fruit growth. *Plant Sci.* 213 18–29. 10.1016/j.plantsci.2013.08.007 24157204

[B42] Sangwan R. Sangwan-Norreel B. (1990). “Genetic transformation and plant improvement,” in *The Impact of Biotechnology on Agriculture*, eds Altman A. Hasegawa P. M. (Berlin: Springer), 299–337.

[B43] Sawa S. Watanabe K. Goto K. Kanaya E. Morita E. H. Okada K. (1999). FILAMENTOUS FLOWER, a meristem and organ identity gene of *Arabidopsis*, encodes a protein with a zinc finger and HMG-related domains. *Genes Dev.* 13 1079–1088. 10.1101/gad.13.9.1079 10323860 PMC316944

[B44] Sherr C. J. Roberts J. M. (1995). Inhibitors of mammalian G1 cyclin-dependent kinases. *Genes Dev.* 9 1149–1163. 10.1101/gad.9.10.1149 7758941

[B45] Sieberer T. Hauser M. T. Seifert G. J. Luschnig C. (2003). PROPORZ1, a putative *Arabidopsis* transcriptional adaptor protein, mediates auxin and cytokinin signals in the control of cell proliferation. *Curr. Biol.* 13 837–842. 10.1016/S0960-9822(03)00327-0 12747832

[B46] Skoog F. Miller C. (1957). Chemical regularion of growth and organ formation in plant fissue cultured. *In vitro. Symp. Soc. Exp. Biol.* 11 118–131. 10.1038/jid.2014.273 13486467

[B47] Ubeda-Tomás S. Federici F. Casimiro I. Beemster G. T. Bhalerao R. Swarup R. (2009). Gibberellin signaling in the endodermis controls *Arabidopsis* root meristem size. *Curr. Biol.* 19 1194–1199. 10.1016/j.cub.2009.06.023 19576770

[B48] Valvekens D. Van Montagu M. Van Lijsebettens M. (1988). *Agrobacterium tumefaciens*-mediated transformation of *Arabidopsis thaliana* root explants by using kanamycin selection. *Proc. Natl. Acad. Sci. U.S.A*. 85 5536–5540. 10.1073/pnas.85.15.5536 16593964 PMC281793

[B49] Wang H. Zhou Y. Gilmer S. Whitwill S. Fowke L. C. (1997). A plant cyclindependent kinase inhibitor gene. *Nature* 386 451–452. 10.1038/386451a0 9087400

[B50] West G. Inzé D. Beemster G. T. (2004). Cell cycle modulation in the response of the primary root of *Arabidopsis* to salt stress. *Plant Physiol.* 135 1050–1058. 10.1104/pp.104.040022 15181207 PMC514139

[B51] Xu K. Liu J. Fan M. Xin W. Hu Y. Xu C. (2012). A genome-wide transcriptome profiling reveals the early molecular events during callus initiation in *Arabidopsis* multiple organs. *Genomics* 100 116–124. 10.1016/j.ygeno.2012.05.013 22664253

[B52] Yamaguchi M. Kato H. Yoshida S. Yamamura S. Uchimiya H. Umeda M. (2003). Control of in vitro organogenesis by cyclin-dependent kinase activities in plants. *Proc. Natl. Acad. Sci. U.S.A.* 100 8019–8023. 10.1073/pnas.1332637100 12799469 PMC164705

[B53] Yasutani I. Ozawa S. Nishida T. Sugiyama M. Komamine A. (1994). Isolation of temperature-sensitive mutants of *Arabidopsis thaliana* that are defective in the redifferentiation of shoots. *Plant Physiol.* 105 815–822. 12232244 10.1104/pp.105.3.815 PMC160727

[B54] Yu Y. Steinmetz A. Meyer D. Brown S. Shen W. H. (2003). The tobacco A-type cyclin. Nicta; CYCA3; 2, at the nexus of cell division and differentiation. *Plant Cell* 15 2763–2777. 14615597 10.1105/tpc.015990 PMC282795

[B55] Zhang Y. Li B. Huai D. Zhou Y. Kliebenstein D. J. (2015). The conserved transcription factors, MYB115 and MYB118, control expression of the newly evolved benzoyloxy glucosinolate pathway in *Arabidopsis thaliana*. *Front. Plant Sci.* 6:343 10.3389/fpls.2015.00343 PMC4429563 26029237

[B56] Zhao X. Y. Su Y. H. Zhang C. L. Wang L. Li X. G. Zhang X. S. (2013). Differences in capacities of in vitro organ regeneration between two *Arabidopsis* ecotypes Wassilewskija and Columbia. *Plant Cell Tiss. Org.* 112 65–74. 10.1007/s11240-012-0216-8

