# Supplementary material for: Down-regulation of multiple CDK inhibitor ICK/KRP genes promotes cell proliferation, callus induction and plant regeneration in Arabidopsis
Source: Front Plant Sci. 2015 Oct 13;6:825. doi: 10.3389/fpls.2015.00825 (PMC4602110; doi:10.3389/fpls.2015.00825)
Supplement: Supplementary file 1 [file Data_Sheet_1.PDF]

*Supplementary Material*

**Down-regulation of multiple CDK inhibitor *ICK/KRP* genes promotes cell proliferation, callus induction and plant regeneration in Arabidopsis**

**Yan Cheng, Han Liu, Ling Cao, Sheng Wang, Yongpeng Li, Yuanyuan Zhang, Wei Jiang, Yongming Zhou* and Hong Wang***

*** Correspondence:** Corresponding Author: hong.wang@usask.ca; ymzhou@mail.hzau.edu.cn

**Supplementary Data:**

- **Supplementary Table 1. Summary of primers used for real-time PCR.**
- **Supplementary Figure 1. Callus induction and growth from cotyledon explants of Wt and *ick1/2/5/6/7* mutant plants.**
- **Supplementary Figure 2. Callus induction from root explants of Wt and *ick1/2/5/6/7* mutant at different concentrations of 2,4-D.**
- **Supplementary Figure 3. Callus induction from cotyledon explants of Wt and *ick1/2/5/6/7* mutant at different concentrations of 2,4-D.**
- **Supplementary Figure 4. Shoot and root regeneration of Wt and *ick1/2/5/6/7*.**
- **Supplementary Figure 5. Root regeneration from root-derived calli of Wt and various *ick* mutant plants.**

**Supplementary Table**

**Supplementary Table 1. Summary of primers used for real-time PCR.**

| Gene name | Gene ID | Primer ID | Primer sequences |
|---|---|---|---|
| *CDKB1;2* | *At2g38620* | *lxp183* | TGGTCTGTTGGATGCATCTTTGCC |
| | | *lxp184* | TGATAAGTCTTGCGGCTCCCACTT |
| *CDKB2;1* | *At1g76540* | *lxp173* | GCTGAACTTGTGACCAACCAAGCA |
| | | *lxp174* | TCCACTGTGGGTATTCATGCCAGT |
| *CYCB1;4* | *At2g26760* | *CYP127* | TAAAGCTGCGGTTCCATGTGATGC |
| | | *CYP128* | AAGGATCTGACGCGCAGCATAAAC |
| *CKS2* | *At2g27970* | *CYP145* | TCGTGGATGGGTTCATTACGCGAT |
| | | *CYP146* | TAGCAATCTGAGCTTGGTGCTCCT |
| *MCM3* | *At5g46280* | *CYP161* | AACACCAAGTGGACGTAGAGGCAA |
| | | *CYP162* | TTCAATCCTTGCTGCAGAGACCGT |
| *MCM4* | *At2g16440* | *CYP163* | ATGCAGCAATCAGCAACGGATCAC |
| | | *CYP164* | AAGGTATCCCGCCTCATTCGTTCA |
| *MCM5* | *At2g07690* | *CYP165* | AAATGAGACTGTCACACGAGGCCA |
| | | *CYP166* | TGATTTCATTGGCCATCTCGCCTG |
| *MCM7/(PRL)* | *At4g02060* | *CYP167* | ATCAGTGGCACAGAGTGATGTGGA |
| | | *CYP168* | TTTCTGGAGATCCAGTTCAGGGCA |
| *PRC1B* | *At4g12620* | *CYP169* | ACAACATTTGACCGAGTGGCAACG |
| | | *CYP170* | GCTGCAGCTTCTGCAATCTATGCT |
| *ICU2* | *At5g67100* | *CYP171* | CTGGCTTAAACTGCATTGCCCGAA |
| | | *CYP172* | TGCATGACTCGTCTTCACACACCA |
| *POLA2* | *At1g67630* | *CYP173* | TCGCTCCTAAGGCGCTACAGATTT |
| | | *CYP174* | GGGTTTACACAAACGCACTTGGCT |
| *PCNA1* | *At1g07370* | *lxp217* | TCGTGAGGATGCCTTCCAATGAGT |
| | | *lxp218* | TGCATCTTCCGGCTTGTCTACAGT |
| *EMB2813* | *At1g67320* | *CYP179* | AGCTCGCATGCACCTTAACCTTTG |

| | | | |
|---|---|---|---|
| | | *CYP180* | TTAAACCGGAGCAGCTGGAGTCTT |
| *POLA3/4* | *At5g41880* | *CYP181* | ACTGCGGACGTGCGTAGAAGAAAT |
| | | *CYP182* | CGCAGAAGGGTGCCTTAAGCAAAT |
| *RNR1* | *At2g21790* | *CYP175* | ACACATGGACAAACCCAACTTCGC |
| | | *CYP176* | ATCGCAGGTAGTACATCCCGGTTT |
| *TSO2* | *At3g27060* | *CYP177* | TGGGCTATGAAATGGATCGACGGT |
| | | *CYP178* | TCCAGGCATGAGTCCTCGTTTCTT |
| *CHR17* | *At5g18620* | *CYP183* | AACTGGATCGCTACAGAAACCCGT |
| | | *CYP184* | AGCTCATCCCAGTTTCCATACCCA |
| *HTA6* | *At5g59870* | *CYP185* | TGCTGGTAACGCTGCGAGAGATAA |
| | | *CYP186* | TGAGCGATTGTGACACCACTCAGA |
| *HTH/EDA17* | *At1g72970* | *CYP187* | AAGTTCTTGGTGTCGACAGGCTCA |
| | | *CYP188* | ATAGTAGCTTGCGGGTTGGTTCCT |
| *BAM3* | *At4g20270* | *CYP189* | AGACAATGGAGCTTCCGAGTGCAT |
| | | *CYP190* | TCCGAAGCTGTACACATCGCTCTT |
| *ANT* | *At4g37750* | *TZ31* | TTAGCGCGAAGGAACAACAACAGC |
| | | *TZ32* | TCCAAGGACTCATATTTCCGCCCA |
| *KLP2* | *At3g44050* | *CYP191* | GAGCAAGGGCGCATGTAAACTCAT |
| | | *CYP192* | ACCCTCTTCCTCATGCCTCTTGTT |
| *KICP-02* | *At3g50240* | *CYP193* | AGAGTGGCAAGCTATGGAAGTGGA |
| | | *CYP194* | ACATTGAGTGTCGGTTCCACGAGT |
| *CCA1* | *At2g46830* | *CYP195* | AAAGGCAAGAGGATGGCACCAATG |
| | | *CYP196* | TGGAGAAGAGAGCTTGGAAGGCAA |
| *ESR1* | *AT1G12980* | *CYP231* | GATCGCTACGGGAATTTTCAAG |
| | | *CYP232* | GCCTAACTGAGTTCCGTACATC |
| *ESR2* | *AT1G24590* | *CYP233* | CCACCGTATTTTCTCGTCTCC |
| | | *CYP234* | TTGACCTCTTAGCTTTAGGCG |
| *CUC1* | *AT3G15170* | *CYP209* | TCAATACCTTTGCGACGGAG |

| | | | |
|---|---|---|---|
| | | *CYP210* | GAAGGAATGTATGAAAGCTCGC |
| *CLE2* | *AT4G18510* | *CYP211* | TTCTGTTATCCTCAATCGCCG |
| | | *CYP212* | TCATCCTCTACAGTCGGACTC |
| *GNAT1* | *AT5G67430* | *CYP213* | CATCAACTCAATCCTCACCAAC |
| | | *CYP214* | ATACTTATAACGGCCCATGACC |
| *WUS* | *AT2G17950* | *CYP215* | GTAGCCATGTCTATGGATCTATGG |
| | | *CYP216* | GACCTTCTAGACCAAACAGAGG |
| *STM* | *AT1G62360* | *CYP217* | TGGAGCCGTCACTACAAATG |
| | | *CYP218* | TCCAATGCCGTTTCCTCTG |
| *PIN1* | *AT1G73590* | *CYP219* | GCAAAGTCTATCTCCATACTCTCAG |
| | | *CYP220* | TCCAACGACAAATCTCATAGCC |
| *REV* | *AT5G60690* | *CYP221* | CCATCCTGTGTTGCTCATTAAAG |
| | | *CYP222* | GAACAGATAGCCTTACGACCC |
| *FIL* | *AT2G45190* | *CYP223* | AAGCCACAGAGAAGCCTTTAG |
| | | *CYP224* | CATCACCATGTTATCCTCTCCC |
| *ATML1* | *AT4G21750* | *CYP235* | GATTCGCTATTTTGCCGGATG |
| | | *CYP236* | GAGTTTAGCGGTAGGAACAGAG |
| *CLV3* | *AT2G27250* | *CYP237* | TGGAAAGTGAATGGGTTGGAG |
| | | *CYP238* | AACTCTTCATGTAGTCCTAAACCC |

Sequences were obtained by SIGnAL Gene iSect Tool (http://signal.salk.edu/isect.2.html#Region), Primers were designed using the IDT DNA Real Time PCR primer design tool (https://www.idtdna.com/scitools/Applications/RealTimePCR/) for amplifying DNA fragments around 100 bp in length, with an annealing temperature about 60°C.

## Supplementary Figures

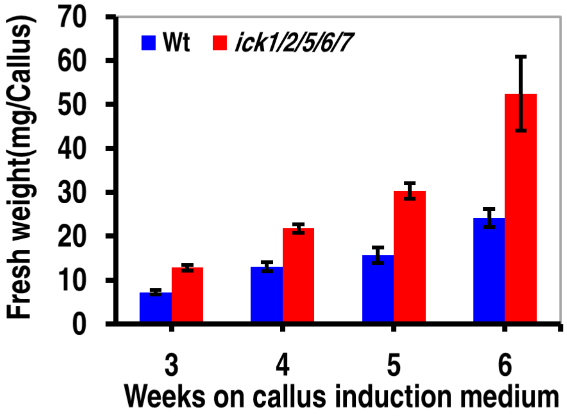


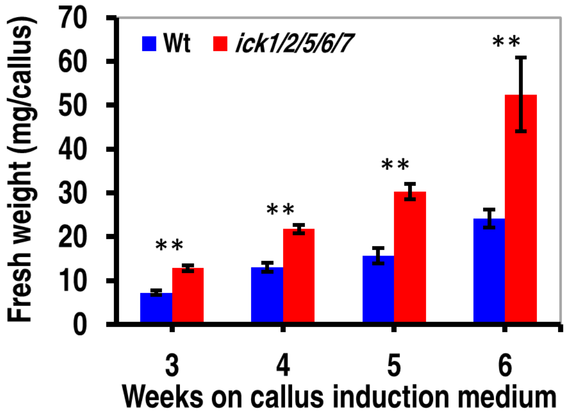


**Supplementary Figure 1. Callus induction and growth from cotyledon explants of Wt and *ick1/2/5/6/7* mutant plants.** The fresh weight of Wt and *ick1/2/5/6/7* mutant calli on the medium containing 2,4-D was measured from the 3rd week to 7th week. For each line, 10 plates were used with each plate having about 15 cotyledon explants. The Student's *t*-test was used for analyzing the significant difference between the Wt and mutant (Error bar=SE; **, $P<0.01$).

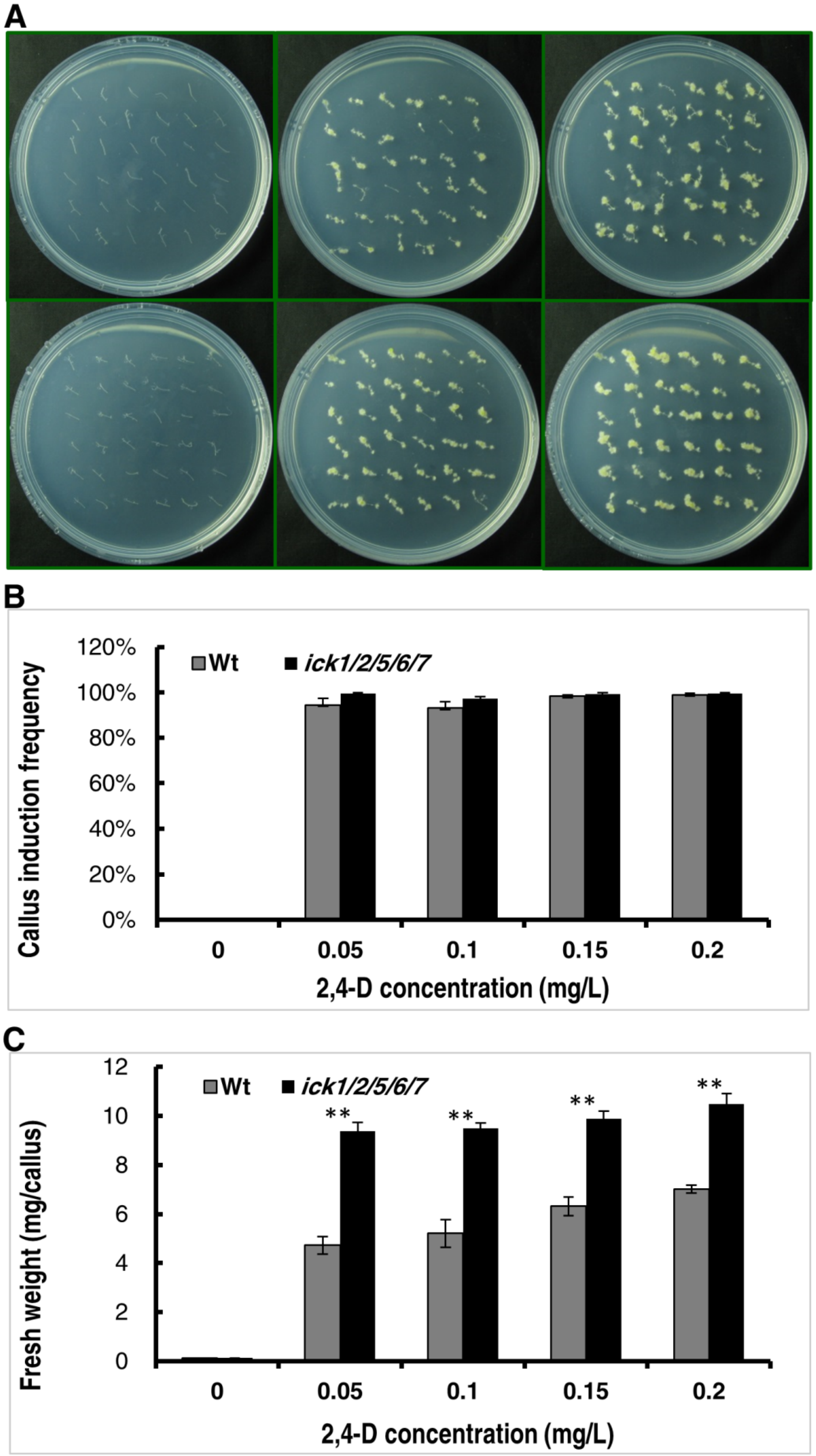


**Supplementary Figure 2. Callus induction from root explants of Wt and *ick1/2/5/6/7* mutant at different concentrations of 2,4-D.** Root explants were cultured on 1/2 MS media containing indicated concentrations of 2,4-D for 20 days. (A) Representative plates showing root explants of Wt

(the upper row) and *ick1/2/5/6/7* mutant (the lower row) on 1/2 MS medium (the 1st column), and 1/2 MS medium containing 0.1 and 0.2 mg/L (the 2nd to 3rd column) 2,4-D. (B) Callus induction frequency of root explants of Wt and *ick1/2/5/6/7* mutant on the indicated media. (C) Fresh weight of root explants with callus of Wt and *ick1/2/5/6/7* mutant after 20 days of culture on indicated medium. The bars show the mean values of 4-5 plates and the error bars show the standard errors. The Student's *t*-test was used for analyzing the significant difference between the Wt and mutant (Error bar=SE; **, P<0.01).

.

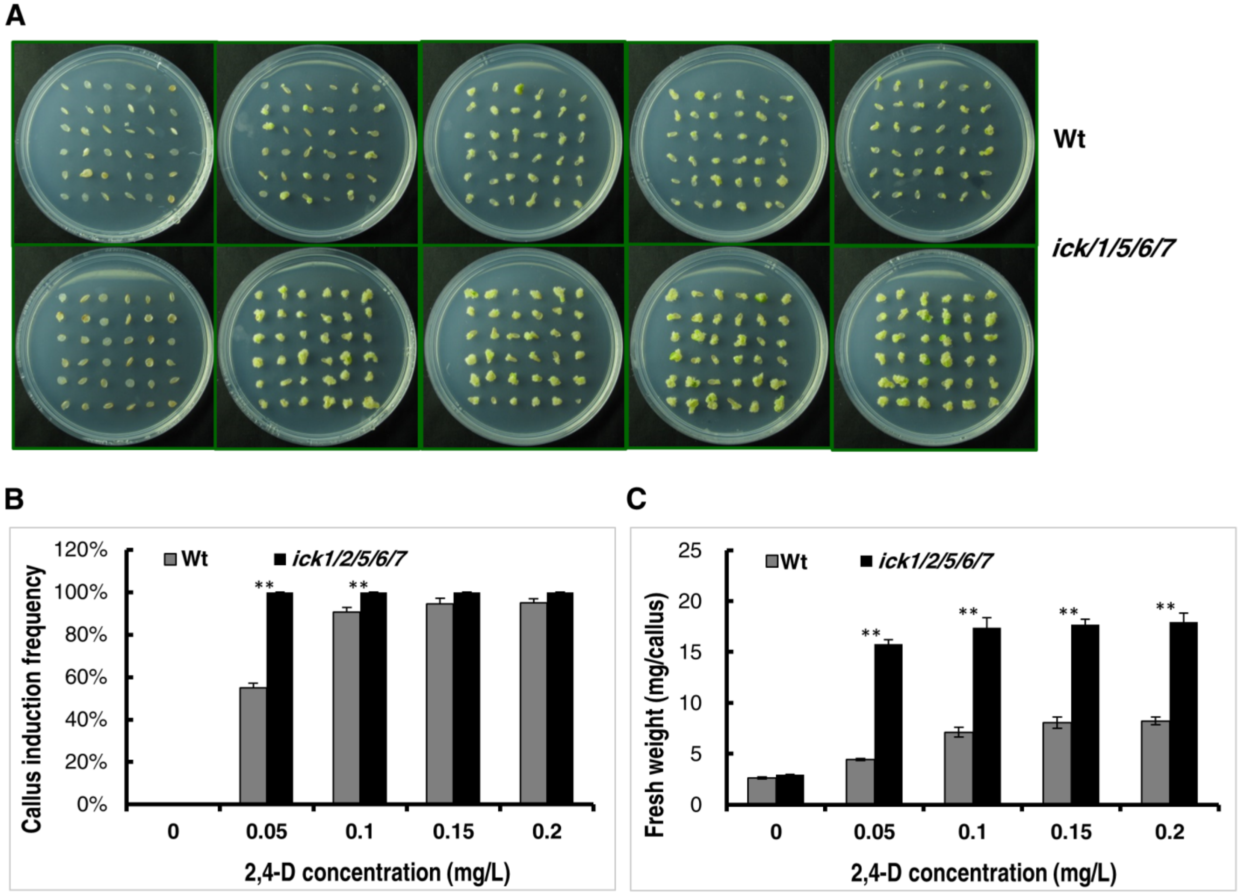


**Supplementary Figure 3. Callus induction from cotyledon explants of Wt and *ick1/2/5/6/7* mutant at different concentrations of 2,4-D.** Cotyledons were excised from 7-day-old seedlings and cultured on the 1/2 MS medium containing indicated concentrations of 2,4-D for 20 days. (A) Representative plates showing cotyledon explants of Wt (the upper row) and *ick1/2/5/6/7* mutant (the lower row) on 1/2 MS medium (the 1$^{st}$ column), and medium containing 0.05, 0.1, 0.15, or 0.2 mg/L (the 2$^{nd}$ to 5$^{th}$column) 2,4-D. (B) Callus induction frequency of the cotyledon explants of Wt and *ick1/2/5/6/7* mutant. (C) Fresh weight of the cotyledon explants with callus of Wt and *ick1/2/5/6/7* mutant after 20 days of culture. The bars show the mean induction rates of 4-5 plates and the error bars show the standard errors. The Student's *t*-test was used for analyzing the significant difference between the Wt and mutant (Error bar=SE; **, $P<0.01$).

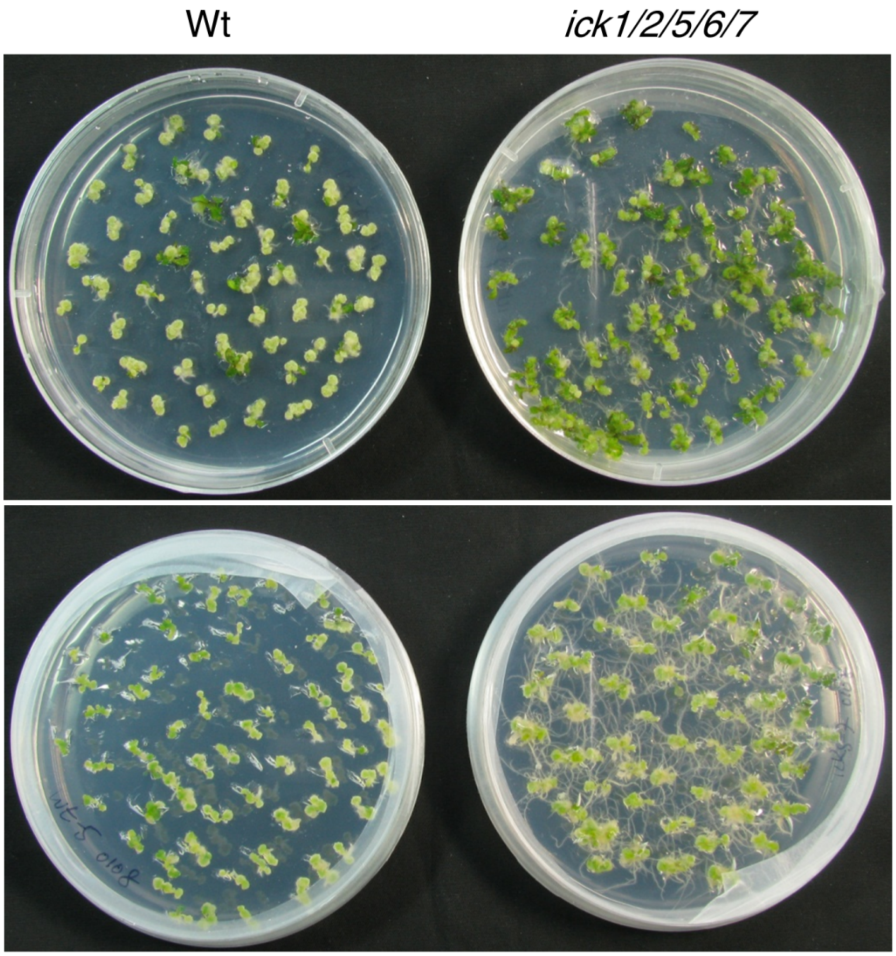


**Supplementary Figure 4. Shoot and root regeneration of Wt (left) and *ick1/2/5/6/7* (right).** Root-derived calli were transferred and cultured on the shoot induction medium (SIM) for 30 days. The first row shows the upside of representative plates and regenerated shoots. The second row shows the downside of the representative plates and regenerated roots on SIM.

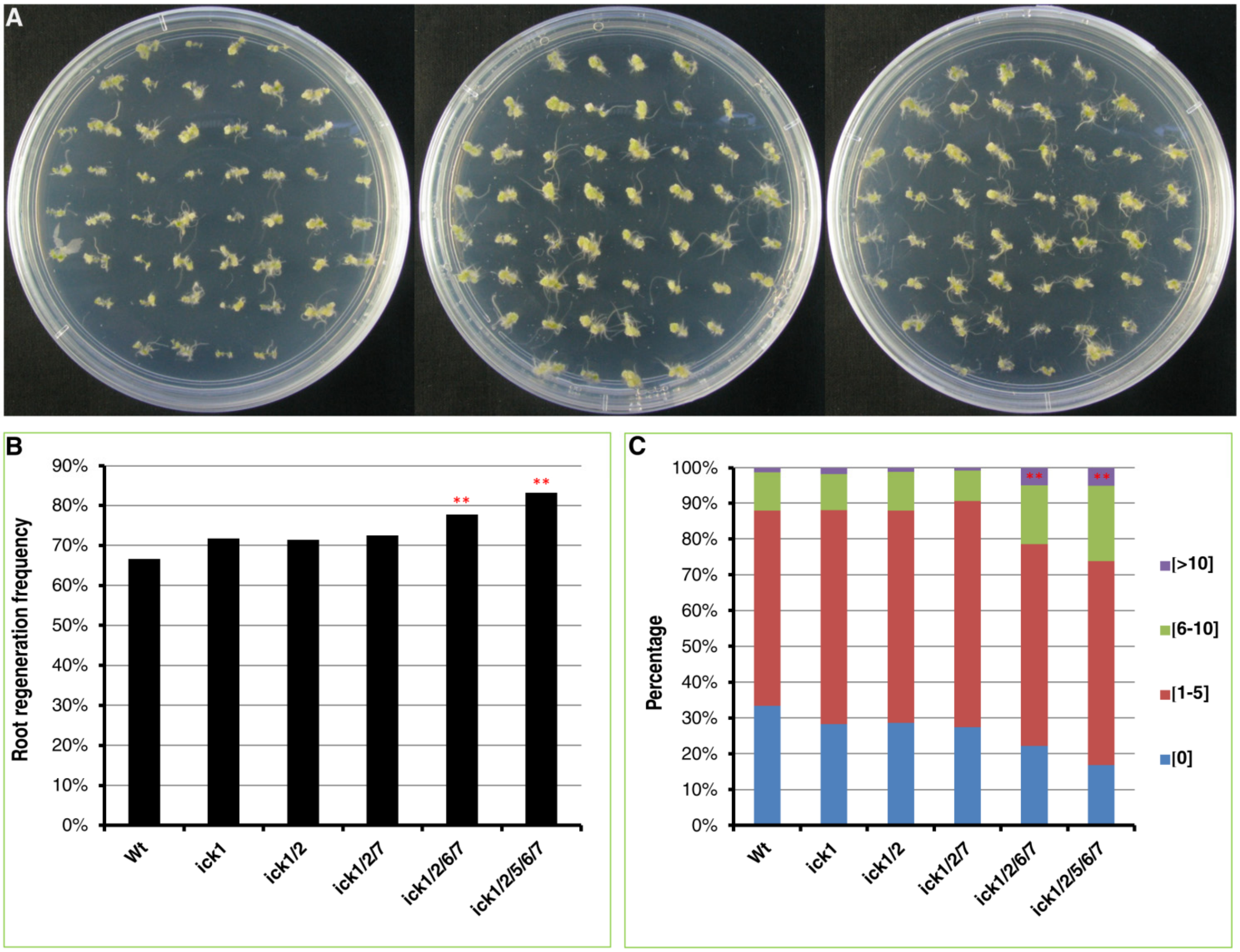


**Supplementary Figure 5. Root regeneration from root-derived calli of Wt and various *ick* mutant plants.** Root explants were cultured on callus induction medium first. After 7 days, explants with callus were transferred onto root induction medium and were cultured for 30 days. For each line, 9-10 plates were used with each plate having about 40 explants. (A) Representative plates showing root regeneration from root-derived calli of Wt (left), *ick1/2/6/7* (middle) and *ick1/2/5/6/7* (right) plants. (B) Frequency of root regeneration for five *ick* mutant and Wt lines. The two-tailed Mann-Whitney U test was applied for analyzing the differential significance of the percentage data. (C) Distribution of calli with different numbers of regenerating roots. Calli are grouped into four types, with 0, 1-5, 6-10, and more than 10 roots on each callus respectively. The two-tailed Mann-Whitney U test was used for analyzing the differences of root regeneration frequency and percentage of >10 roots calli between the Wt and each mutant (**, $P<0.01$).
